# mRNA quantification and clinical evaluation of telomerase reverse transcriptase subunit (hTERT) in intracranial tumours of patients in the island of Crete

**DOI:** 10.1038/sj.bjc.6602642

**Published:** 2005-06-28

**Authors:** A Yannopoulos, E Dimitriadis, A Scorilas, T Trangas, E Markakis, M Talieri

**Affiliations:** 1 Department of Neurosurgery, University Hospital of Heraclio, Heraclio, Crete, Greece; 2 Department of Genetics, Saint Savas Hospital, Athens, Greece; 3 Department of Biochemistry and Molecular Biology, Faculty of Biology, University of Athens, Athens 15711, Greece; 4 ‘G. Papanicolaou’ Research Center of Oncology, ‘Saint Savas’ Hospital, Athens 11522, Greece

**Keywords:** brain tumours, hTERT, real-time PCR, malignant gliomas, meningiomas, prognosis

## Abstract

Telomerase is a reverse transcriptase that maintains telomeres by adding telomeric TTAGGG repeats to the ends of human chromosomes. The aim of this study was to evaluate quantitatively the mRNA expression of telomerase catalytic subunit (hTERT) in different types of intracranial tumours in relation to their histologic pattern and grade and correlate it with progression-free (PFS) and overall survival (OS) of patients. Human telomerase reverse transcriptase mRNA levels were estimated by the use of real time RT–PCR in 68 samples of intracranial tumours. It revealed statistical correlation between hTERT mRNA expression levels and the grade of the tumours (*P*<0.001). Patients having negative expression of hTERT mRNA had statistically longer PFS (*P*=0.031) and OS (*P*=0.047). Cox univariate regression analysis revealed that hTERT mRNA-positive patients had a high and statistically significant risk of relapse (hazard ratio (HR) of 2.24 and *P*=0.038). In the Cox multivariate regression model, the levels of hTERT mRNA were adjusted for tumour grade and patients age, and since there was statistically significant relationship between the levels of hTERT mRNA and the grade of the tumours (*P*=0.003 or *P*=0.006, respectively), hTERT mRNA levels could not be considered as an independent prognostic factor for PFS or OS.

Most eukaryotic cells that can divide *in vivo* cannot do so indefinitely. The process that limits the proliferative potential of cells has been termed cellular or replicative senescence. The mechanism by which cells sense the number of divisions they have completed appears to depend, at least in part, on the length of their telomeres (Campisi, 1997). Telomeres are long repetitive nucleotide sequences at both ends of chromosomes that consist of hundreds to thousands of tandem repeats of the sequence TTAGGG. Their primary role is to protect chromosome ends from recombination, fusion and DNA degradation. Telomeres shorten progressively with each round of cell division as a result of the inability of DNA polymerases to replicate the 5′ end of linear DNA, and erosion of these sequences beyond a critical point is thought to signal cell cycle arrest and entry into cellular senescence (O'Reilly *et al*, 1999). Telomerase is a ribonucleoprotein polymerase composed of an integral RNA component (hTR) (Feng *et al*, 1995) and several protein components, including the reverse transcriptase subunit human telomerase reverse transcriptase (hTERT) (Kilian *et al*, 1997; Nakayama *et al*, 1998). The RNA component hTR contains the template for the reverse transcription of telomeres, and hTERT is the reverse transcriptase that catalyses this reaction. The RNA component hTR and hTERT are indispensable for telomerase activity, and expression of these subunits *in vitro* leads to the formation of a functional telomerase enzyme (Weinrich *et al*, 1997; Counter *et al*, 1998; Nakayama *et al*, 1998). In vertebrate organisms, telomerase activation is a mechanism known to stabilise the loss of telomeres. Most somatic cells, regardless of their rate of proliferation undergo a steady-state rate of telomere loss and do not posses detectable telomerase activity. In contrast, all immortal cells examined to date show no loss of telomere length or sequence with division, suggesting that maintenance of telomeres is required for cells to escape from replicative senescence and proliferate indefinitely (Kim, 1997; Dhaene *et al*, 2000). Recent studies in which the gene encoding hTERT was introduced into normal human cells that lack hTERT resulted in reconstitution of telomerase activity, maintenance of telomeres, extension of replicative capacity and transformation of normal cells into cancer cells (Hahn *et al*, 1999). In humans, telomerase has been detected in most types of cancer, including those of the brain (Kotoula *et al*, 2004; Novakovic *et al*, 2004; Snuders *et al*, 2004), suggesting a role for telomerase in sustaining proliferative capacity to cells, which is a requirement for progression towards malignancy. It is usually not detected in normal somatic cells, except for proliferating stem cells, during embryonic development, and in adult germline tissues.

Malignant gliomas account for the largest number of primary brain tumours in adults. Even with best conventional therapy, the prognosis is poor. Most patients do not survive beyond 1 year after the diagnosis of glioblastoma multiforme, for more than 5 years with anaplastic astrocytoma, and usually for not more than 10 years with oligodendroglioma. Brain tumours were one of the first systems in which telomerase activity was assessed as a marker of malignancy (Langford *et al*, 1995). To date, it is generally accepted that high telomerase activity levels characterise high-grade gliomas (Le *et al*, 1998; Falchetti *et al*, 1999; Huang *et al*, 1999; Harada *et al*, 2000; Sugita *et al*, 2000); however, there is no agreement on the distinctive or prognostic value of this parameter (Kleinschmidt-Demasters *et al*, 2000), while telomerase positivity rates vary extensively among the different studies (23.6–100%) (Langford *et al*, 1995; Chong *et al*, 1998; Le *et al*, 1998; Falchetti *et al*, 1999; Huang *et al*, 1999; Harada *et al*, 2000; Kleinschmidt-Demasters *et al*, 2000; Sugita *et al*, 2000).

Meningiomas are estimated to constitute up to 25% of primary intracranial tumours (Kleihues and Cavenee, 2000). They are typically benign tumours, but the incidence of growth at inoperable sites and recurrence complicate surgical management. The limited understanding of aetiology and growth of these tumours has hindered the development of an adjuvant therapy. The hypotheses that reactivation of telomerase is required to maintain tumour growth (Kim *et al*, 1994) or may play a critical role in the progression or maintenance of the malignant phenotype (Rhyu, 1995) are in conflict with findings in benign meningiomas. Several studies have reported that telomerase activity is present in the vast majority of malignant brain tumours, while enzyme activity could not be detected in benign meningiomas (Carroll *et al*, 1999; Falchetti *et al*, 1999; Hiraga *et al*, 1998; Sano *et al*, 1998; Cabuy and de Ridder 2001; Boldrini *et al*, 2003).

In the present study, the expression level of hTERT mRNA was determined by the method of real-time quantitative RT–PCR in a cohort of 68 intracranial tumours, consisting of 34 malignant gliomas, 18 meningiomas, seven metastatic tumours to the brain and seven miscellaneous intracranial tumours. Real-time quantitative RT–PCR methodology is a modern technique that can provide significant and quantitative information about gene transcripts in an automated, rapid, versatile and cost-effective way. Furthermore, the present study examines exclusively Greek population leaving permanently through out their lives at the island of Crete.

## MATERIALS AND METHODS

### Patients and samples

The 68 brain tumour samples used in this study were resected surgically from 67 patients at the Department of Neurosurgery of the University Hospital of Heraclion, Crete, between 1999 and 2003. Follow-up information was available for 60 of them. Histologic diagnoses and grading of tumours were made based on the revised World Health Organization (WHO) classification of brain tumours (WHO, 2000). A total of 36 samples were neuroepithelial tumours (17 glioblastomas multiforme (grade IV); eight astrocytomas (five grade I, one grade II, two grade III,); two myeloblastomas (grade IV); three oligodendrogliomas (grade III); two ependymomas (one grade I and one grade II); four neurinomas (one central neurinoma grade I and three acoustic neurinomas, grade I)); 18 samples were meningeal tumours (meningiomas); seven miscellaneous brain tumors (one pituitary adenoma; one non-Hodgkin B-lymphoma; one osteoma; one cholosteatoma; one hemangioblastoma; one choroid plexus papilloma; one nonspecific inflammation) and seven metastatic brain tumours (four from lung adenocarcinoma, two from breast adenocarcinoma and one from melanoma) (Table 1). For a recurrent glioblastoma multiform case, both primary and recurrent tumour was examined. In addition, adjacent normal brain tissues, which were obtained by corticotomy while attempting to reach the tumours of two primary glioblastomas multiform patients, were used as controls. The controls were sufficiently separated from the tumours and appeared normal to the naked eye. All cases under study came from total surgical removal. No chemotherapy or radiotherapy was administered before surgery. Investigations were carried out in accordance with the ethical standards of the Helsinki Declaration of 1975, as revised in 1983. Informed consent was obtained from all patients for the scientific analysis of tumour samples.

Specimens for the analysis of hTERT and histologic observation were taken from the same general region of the tumour during surgery. Immediately after surgery, the samples were snap-frozen and stored in liquid nitrogen until RNA extraction. Furthermore, the patient with the recurrent glioblastoma multiforme was previously irradiated locally with a dose of 60 grays (Gy) and given 3 mg m^−2^ dose of cisplatin. All patients with primary glioblastoma multiforme and anaplastic astrocytoma also underwent irradiation and were given cisplatin (2–4 mg m^−2^) after surgery.

### Total RNA isolation

Total RNA isolation was performed using the Trizol LS reagent (Invitrogen; Carlsbad, CA, USA) according to the manufacturer's instructions. All of the preparation and handling steps of RNA took place in a laminar flow hood, under RNAse-free conditions. The isolated RNA was dissolved in RNA storage buffer (Ambion; Houston, TX, USA) and stored at −80°C until used. RNA concentration was determined by absorbance readings at 260 nm with the HITACHI UV-VIS (U-2000) spectrophotometer. RNA integrity was tested by PCR amplification of the b-actin housekeeping gene, as we described previously (Spiropoulou *et al*, 2004).

### Real-time RT–PCR for hTERT mRNA quantification

Human telomerase reverse transcriptase mRNA was quantificated by the use of the LightCycler, Telo TAGGG hTERT quantification kit on a Light Cycler (both from Roche Molecular Biochemicals; (Mannheim, Germany) according to the manufacturer's instructions.

**Figure 1:**
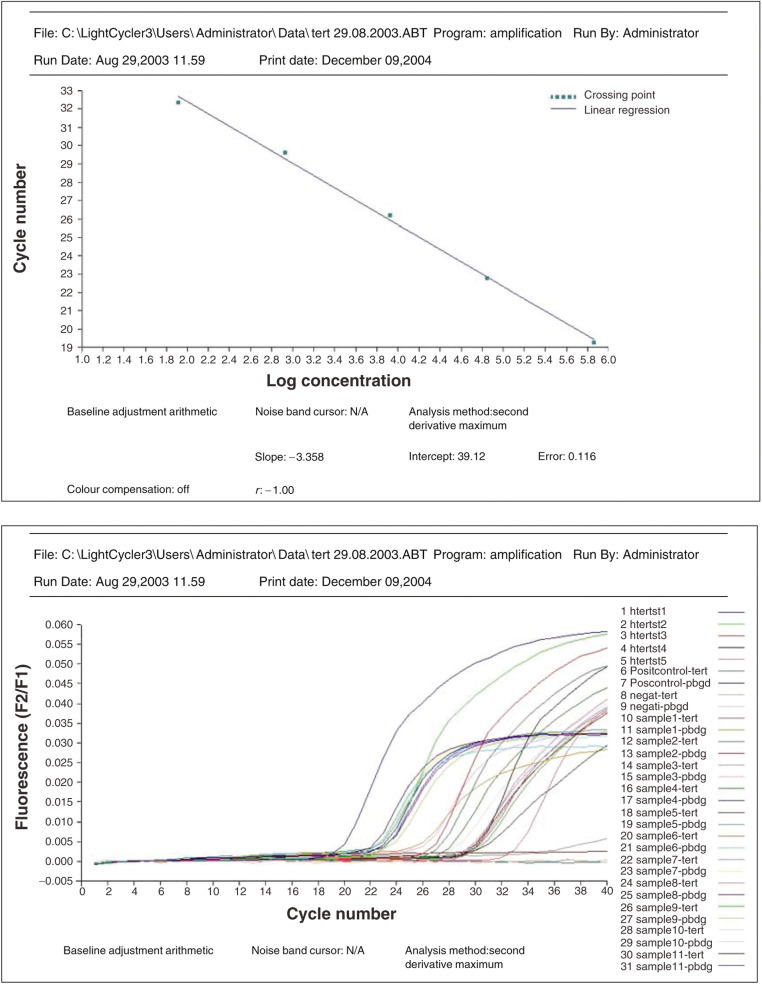
Example of real-time RT–PCR quantification of hTERT mRNA in tumour samples. Top, hTERT standard curve; bottom, hTERT analysis (htertst1, 2, 3, 4, 5 are human TERT RNA Standards with concentration of 7.3 × 10^5^, 7.0 × 10^4,8^, 5 × 10^3^, 8.4 × 10^2^, 8.1 × 10^1^ copies per 2 *μ*l, respectively; Positcontrol=positive control RNA; negat=negative control; samples 1–11 correspond to intracranial tumour samples).

In short description, telomerase (hTERT)-encoding mRNA was reversed transcribed and a 198 bp fragment (corresponding to the full-length hTERT transcript) of the generated cDNA was amplified with specific primers in a one-step RT–PCR reaction. The amplicon was detected by fluorescence using a specific pair of hybridisation probes. The hybridisation probes consist of two different short oligonucleotides that hybridise to an internal sequence of the amplified fragment during the annealing phase of the amplification cycle. One probe is labelled at the 5′-end with LightCycler-Red 640, and to avoid extension, modified at the 3′-end by phosphorylation. The other probe is labelled at the 3′-end with fluorescein. The emitted fluorescence of LightCycler-Red 640 was then measured at the instrument LightCycler. In a separate one-step RT–PCR, mRNA, encoding for porphobilinogen deaminase (PBGD), was processed for use as a house-keeping gene. The reaction product served both as a control for RT–PCR performance and as a reference for relative quantification. During initial optimisation of PCR conditions, amplified products were analysed using agarose gels to ensure correct product size. Once the PCR product was determined with Light Cycler melting-curve analysis, it was used to control for the specificity of amplifications. The number of transcripts in samples was calculated with the LightCycler software, using the calibration data obtained from serial dilutions of purified PCR products containing known numbers of cDNA molecules of each gene (Figure 1). The results of real-time PCR were given as the ratio between hTERT and PBGD transcripts, expressed as a percentage and converted to arbitrary units dividing by 1000. All experiments were performed in duplicate, with good consistency of results (the mean coefficient of variation was 9.5%). All samples were analysed in a blind-trial fashion.

### Statistical analysis

A Cox proportional hazard regression model (Cox, 1972) was developed to evaluate the association (i.e. hazard ratio and its confidence interval) between the potential prognostic marker and progression-free (PFS) or overall survival (OS). This analysis was conducted at both univariate and multivariate levels. The multivariate model was adjusted for hTERT mRNA expression in tumours, tissue grade and patients age. Survival analysis was performed by constructing Kaplan–Meier PFS and OS curves (Kaplan and Meier, 1958) for hTERT-positive and hTERT-negative patients, and the log-rank test was used to compare survival between subgroups of patients. Progression-free survival was defined as the time interval between the date of surgery and the date of identification of recurrent or metastatic disease. Overall survival was defined as the time interval between the date of surgery and the date of death. Other analyses of statistical links between biological and clinical parameters were performed using standard tests.

## RESULTS

**Figure 2:**
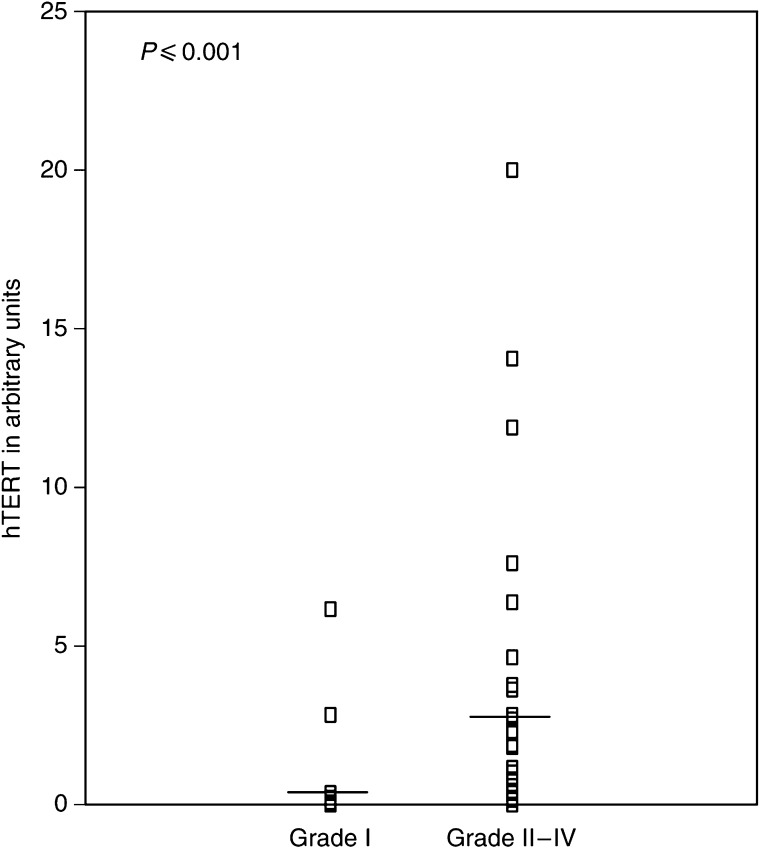
Association of hTERT mRNA expression, defined in arbitrary units, with tumour grade. *P*-value was calculated by Mann–Whitney test. Horizontal lines represent the mean.

**Figure 3:**
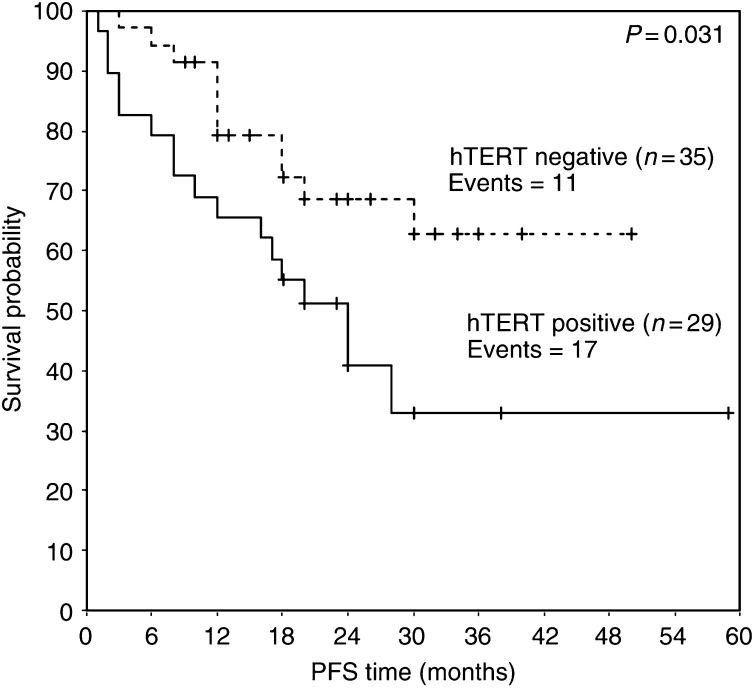
Kaplan–Meier curve for PFS of patients with hTERT mRNA-positive and hTERT mRNA-negative intracranial tumours.

**Figure 4:**
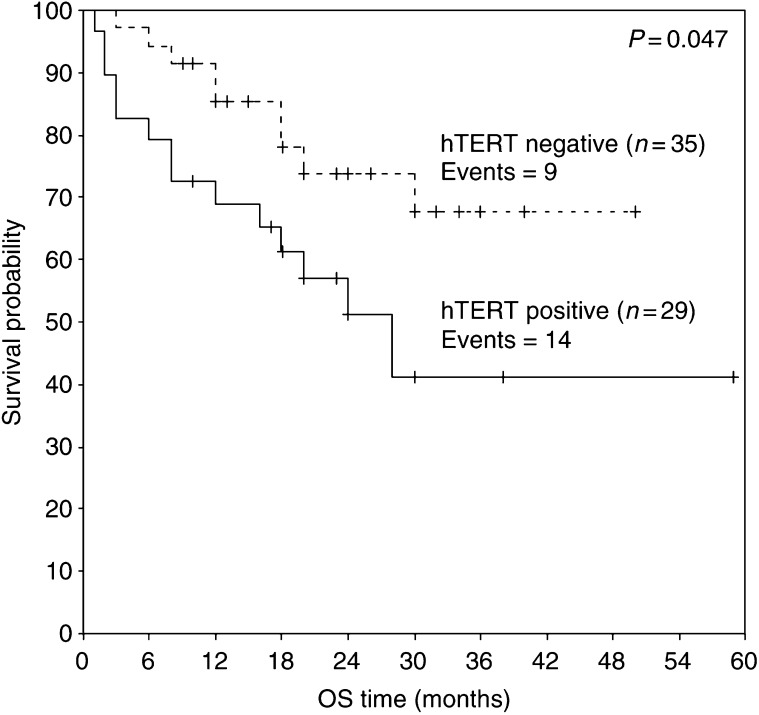
Kaplan–Meier curve for OS of patients with hTERT mRNA-positive and hTERT mRNA-negative intracranial tumours.

We studied 68 intracranial tumours for the presence of hTERT mRNA using a real-time RT–PCR. Table 1 presents the classification of the samples examined in the study according to WHO histologic criteria and the percentage of detectable hTERT mRNA expression in them. The percentage of patients with detectable hTERT mRNA expression is markedly increased with enhanced malignancy (*P*<0.001, Table 4): hTERT mRNA expression was detected in eight out of 33 samples of low malignancy (24.2%), in 19 out of 27 samples of high malignancy (70.4%) and in five out of seven samples metastatic to the brain (71.4%) (Table 2). Patient age ranged from 1 to 76 years with a mean of 48.6±2.54. human telomerase reverse transcriptase arbitrary units ranged from 0.018–19.97 with a mean of 1.44±0.42 (Table 3). The detection limit of hTERT was found to be 0.036 arbitrary units and half of this value (0.018 arbitrary units) was used for the estimation of negative values for hTERT mRNA. Enlistment of samples as primary-low malignant, primary-high malignant and metastatic-high malignant and correlation with hTERT mRNA arbitrary units expression showed statistical significance (*P*<0.001) (Table 4) calculated by the Kruskal–Wallis test. Figure 2 shows that there is statistically significant correlation (*P*<0.001) between hTERT mRNA expression and the grade of the tumours. Progression-free survival probability is significantly lower for patients having positive hTERT expression (*P*=0.031) (Figure 3). Overall survival is significantly lower for patients with positive hTERT expression (*P*=0.047) (Figure 4). Cox univariate regression analysis shows that hTERT-positive patients had a high and statistically significant risk for relapse (hazard ratio (HR)=2.24, *P*=0.038, respectively) (Table 5). Human telomerase reverse transcriptase values were adjusted for tumour grade and patients age in order to construct the Cox multivariate regression model. Since statistically significant relationship was found between hTERT mRNA and tumour grade (*P*<0.001), hTERT mRNA could not be considered as an independent prognostic factor for PFS or OS of patients having intracranial tumours.

## DISCUSSION

It seemed reasonable that telomerase expression might be a useful new prognostic marker in brain tumours, based on numerous studies in the literature on its prognostic implications in other tumour types, like breast, colorectal, endometrial, cervical or ovarian cancer. These studies come to diverse conclusions as to whether telomerase expression has prognostic utility and emphasise that the question needs to be addressed for each individual tumour type. The present study examines intracranial tumours of different histotypes as they presented randomly to the Neurosurgery Department of the University Hospital of Heraclio at the island of Crete, between the years 1999 and 2003. Most previous studies for hTERT expression have employed qualitative rather than quantitative methods. In regard to brain tumours, Langford *et al* (1997) observed a highly significant correlation in ordinary meningiomas between the presence of telomerase activity and a poor prognosis for the patient, a finding that was corroborated by Falchetti *et al*, 1999. In line with this, Boldrini *et al* (2003) reported a significant association between telomerase activity and hTERT mRNA expression, both tending to increase as the histologic grading of meningiomas increased, suggesting a role of telomerase reactivation in the progression of these tumours. In the present study, four out of 18 meningiomas and three out of eight grade I meningiomas were positive for hTERT mRNA expression (data not shown), suggesting also a trend for increased expression with histologic grading of meningiomas. On the other hand, studies by Falchetti *et al* (1999), Harada *et al* (2000), Cabuy and de Ridder (2001) and Tchirkov *et al* (2003) have generated conflicting results regarding the prognostic utility of telomerase expression in meningiomas and gliomas (predominantly of high grade).

In this study, we measured the hTERT mRNA level in 68 intracranial tumours using a recently introduced real-time quantitative PCR technique. We found a progressive increase in the hTERT detection rate with increasing levels of malignancy (*P*<0.001) (Table 4). Present data are coarsely in agreement with the detection rates of telomerase activity reported for similar tumour types (DeMasters *et al*, 1997; Hiraga *et al*, 1998; Le *et al*, 1998; Harada *et al*, 2000; Sugita *et al*, 2000). The correlation of hTERT mRNA expression with the level of malignancy is highly specific when comparing as a group the more aggresssive tumours (gliomas and metastatic tumours) and tumours regarded usually as benign (meningiomas, neurinomas) (*P*=0.001) (Table 4). This association is in line with the demonstration of a similar association at the level of hTERT-protein overexpression (Chakravarti *et al*, 2001). Among patients with detectable levels of hTERT mRNA, the amount of hTERT transcripts was significantly higher among glioblastoma patients as well as patients with metastatic tumours to the brain in comparison to patients with other tumour histotypes (data not shown). Unfortunately, the small number of samples present in each category does not allow separate statistical correlation for patients operated for neuroepithelial, meningial or metastatic tumours with their survival. Nevertheless, not all glioblastomas or all of metastatic tumours to the brain were found to express hTERT transcripts. Similar results have been reported from other authors. Tchirkov *et al* (2003), using the same method, reported hTERT mRNA-positive expression in 69.8% of glioblastoma multiforme samples, when in the present study we found 66.6%. Kleinschmidt-DeMasters *et al* (2000) analysed by the TRAP method and found hTERT expression in 89% of glioblastoma samples. All of those results are discordant with the conclusion of Falchetti *et al* (1999) that the assessment of telomerase activity influence tumour prognosis in malignant gliomas. Kleinschmidt-DeMasters *et al* (2000) addressed this question by analysing quantitatively two to three regions from each tumour and found three-fold regional variation in telomerase levels within the same tumour. They concluded that even in glioblastomas that show positivity for telomerase expression, there is variability in the level of telomerase expression from region to region within the tumour. They observed that areas of tissue were negative for telomerase expression when they contained small number of tumour cells. Some negative telomerase-expressing glioblastoma or metastatic samples of our study possibly are taken from infiltrating edge of tumours, where smaller numbers of tumour cells were present relative to reactive or normal tissue or from areas with extensive necrosis. Two out of the seven metastatic brain tumours examined in the present study were negative for hTERT mRNA expression, whenever the positive samples showed a great variability in the expression levels of hTERT mRNA. The time interval to demise for all patients with metastatic tumours was from 6 to 12 months (data not shown). Although the number of metastatic brain tumours in the present study is small, there is a similarity with the results of Kleinschmidt-DeMasters *et al* (1998) reporting a four-fold logarithmic variability but no correlation with tumour type or interval to demise.

In conclusion, the present study revealed statistical correlation between hTERT mRNA expression levels and the grade of the tumours (examined regardless of their histotypes) (*P*<0.001) (Figure 2). In our study, patients of all histological types having negative expression of hTERT mRNA had statistically longer PFS (*P*=0.031) (Figure 3) and OS (*P*=0.047) (Figure 4). Cox univariate regression analysis showed that hTERT mRNA-positive patients had a high and statistically significant risk of relapse (HR of 2.24 and *P*=0.038) (Table 5). In the Cox multivariate regression model, the levels of hTERT mRNA were adjusted for tumour grade and patients age. Since there was statistically significant relationship between the levels of hTERT mRNA and the grade of the tumours (*P*=0.003 or *P*=0.006, respectively), hTERT mRNA levels could not be considered as an independent prognostic factor for PFS or OS.

## Figures and Tables

**Table 1:** human telomerase reverse transcriptase (hTERT)-positive expression in 68 intracranial tumours

**Histology (WHO grade)**	***n***	**+ hTERT expression (*n*)**	**%**
*Neuroepithelial tumours*			
Glioblastoma grade IV	17	10	58.8
Myeloblastoma grade IV	2	2	100
Astrocytoma grade III	2	2	100
Astrocytoma grade II	1	1	100
Astrocytoma grade I	5	1	20
Oligodendroglioma grade III	3	2	66.6
Ependymoma grade I	1	0	0
Ependymoma grade II	1	1	100
Neurinoma grade I	4	1	25
			
*Meningeal tumours*			
Meningioma grade I	18	4	22.22
			
*Miscellaneous tumours of the brain*			
Cholosteatoma grade I	1	1	100
Lipoma	1	1	100
Osteoma, benign	1	0	0
B-lymphoma non-Hodgkin	1	1	100
Hemaglioblastoma	1	0	0
Choroid plexus papilloma	1	0	0
			
*Metastatic brain tumours from other tissues*			
From breast	2	2	100
From lung	4	2	50
From melanoma	1	1	100
			
			
*Nonspecific inflammation*	1	0	0
*Non-neoplastic brain specimens*	2	0	0

**Table 2:** Classification of tumours examined for human telomerase reverse transcriptase (hTERT) mRNA expression according to their histologic malignancy status

	**No. of samples**	**+ hTERT mRNA expression**
*Low malignancy*		
Meningiomas	18	4
Neurinomas	4	1
Ependymoma (grade I)	1	0
Astrocytomas (grade I)	5	1
Haemaglioblastoma	1	0
Osteoma	1	0
Lipoma	1	1
Cholosteatoma	1	1
Choroid plexus papilloma	1	0
		
*Total*	*33*	*8*
		
*High malignancy*		
Glioblastoma	17	10
Myeloblastoma	2	2
Ependymoma (grade II)	1	1
Astrocytoma (grade II or III)	3	3
B-lymphoma non-Hodgkin	1	1
Oligodendroglioma	3	2
		
*Total*	*27*	*19*
		
*Nonspecific inflammation*	1	0
*Metastatic from other tissues*	*7*	*5*

**Table 3:** Descriptive statistics of continuous variables of the study

			**Percentiles**				
			**10**	**25**	**50**	**75**	**90**
**Variable**	**Mean±s.e.^a^**	**Range**	**(Median)**				
hTERT (arbitrary units)	1.44±0.42	0.018–19.97	0.018	0.018	0.036	0.88	4.64
Age (years)	48.61±2.54	1–76	13.8	32.2	54.5	65.7	73.1

a Standard error.

**Table 4:** Human telomerase reverse transcriptase (hTERT) mRNA (arbitrary units) distribution in low and high malignant potential brain tumours

**Tumour**	**Mean±s.e.^a^**	**Median**	**Range**	***P***-**value^b^**
Primary low malignant brain tumours (*N*=33)	0.31±0.20	0.017	0.018–6.13	
Primary high malignant brain tumours (*N*=27)	2.11±0.68	0.37	0.018–14.06	<0.001
Metastatic high malignant brain tumours (*N*=7)	4.54±2.76	1.16	0.018–19.97	

a Standard error.

b Calculated by Kruskal–Wallis test.

**Table 5:** Univariate and multivariate analysis of human telomerase reverse transcriptase (hTERT) mRNA status with regard to progression-free and overall survival

	**Progression-free survival**			**Overall survival**		
	**HR^a^**	**95% CI^b^**	***P*-value**	**HR^a^**	**95% CI^b^**	***P*-value**
**Variable**	**Univariate analysis**					
*hTERT mRNA*						
Negative	1.00			1.00		
Positive	2.24	1.046–4.97	0.038	2.26	0.97–5.24	0.057
log hTERT(continuous variable)	1.51	1.081–2.13	0.016	1.44	0.99–2.11	0.056
Grading (ordinal)	2.49	1.68–3.69	<0.001	2.38	1.57–3.61	<0.001
Age	1.02	1.00–1.04	0.031	1.03	1.00–1.05	0.022
						
	**Multivariate analysis^c^**					
*hTERT mRNA*						
Negative	1.00			1.00		
Positive	0.71	0.31–1.63	0.42	0.83	0.33–2.04	0.68
hTERT (continuous variable)	1.05	0.91–1.11	0.92	0.99	0.89–1.10	0.84
Grading (ordinal)	2.51	1.64–3.81	<0.001	2.34	1.51–3.65	<0.001
Age	1.02	0.99–1.05	0.075	1.03	1.00–1.06	0.028

a Hazard ratio (HR) estimated from Cox proportional hazard regression model.

b Confidence interval of the estimated HR.

c Multivariate models were adjusted for tumour grade and patient age.
